# Analysis of the functional capacity outcome measures for myotonic dystrophy

**DOI:** 10.1002/acn3.50845

**Published:** 2019-07-22

**Authors:** Aura Cecilia Jimenez‐Moreno, Nikoletta Nikolenko, Marie Kierkegaard, Alasdair P. Blain, Jane Newman, Charlotte Massey, Dionne Moat, Jas Sodhi, Antonio Atalaia, Grainne S. Gorman, Chris Turner, Hanns Lochmüller

**Affiliations:** ^1^ John Walton Muscular Dystrophy Research Centre, Institute of Genetic Medicine Newcastle University Newcastle upon Tyne UK; ^2^ Welcome Trust Mitochondrial Research Centre, Institute of Neurosciences Newcastle University Newcastle upon Tyne UK; ^3^ National Hospital for Neurology and Neurosurgery University College London Hospitals NHS Foundation Trust London UK; ^4^ Division of Physiotherapy, Department of Neurobiology, Care Sciences and Society Karolinska Institutet Stockholm Sweden; ^5^ Functional Area Occupational Therapy & Physiotherapy, Allied Health Professionals Function Karolinska University Hospital Stockholm Sweden; ^6^ The Newcastle upon Tyne Hospitals NHS Foundation Trust Newcastle upon Tyne UK; ^7^ Center of Research in Myology Sorbonne Université Paris France; ^8^ Department of Neuropediatrics and Muscle Disorders, Medical Center, Faculty of Medicine University of Freiburg Freiburg Germany; ^9^ Centro Nacional de Análisis Genómico (CNAG‐CRG), Center for Genomic Regulation Barcelona Institute of Science and Technology (BIST) Barcelona Spain; ^10^ Research Institute The Children’s Hospital of Eastern Ontario Ottawa Canada; ^11^ Division of Neurology, Department of Medicine Ottawa University Ottawa Canada

## Abstract

**Objectives:**

Defining clinically relevant outcome measures for myotonic dystrophy type 1 (DM1) that can be valid and feasible for different phenotypes has proven problematic. The Outcome Measures for Myotonic Dystrophy (OMMYD) group proposed a battery of functional outcomes: 6‐minute walk test, 30 seconds sit and stand test, timed 10 m walk test, timed 10 m walk/run test, and nine‐hole peg test. This, however, required a large‐scale investigation,

**Methods:**

A cohort of 213 patients enrolled in the natural history study, PhenoDM1, was analyzed in cross‐sectional analysis and subsequently 98 patients were followed for longitudinal analysis. We aimed to assess: (1) feasibility and best practice; (2) intra‐session reliability; (3) validity; and (4) behavior over time, of these tests.

**Results:**

OMMYD outcomes proved feasible as 96% of the participants completed at least one trial for all tests and more than half (*n* = 113) performed all three trials of each test. Body mass index and disease severity associate with functional capacity. There was a significant difference between the first and second trials of each test. There was a moderate to strong correlation between these functional outcomes and muscle strength, disease severity and patient‐reported outcomes. All outcomes after 1 year detected a change in functional capacity except the nine‐hole peg test.

**Conclusions:**

These tests can be used as a battery of outcomes or independently based on the shown overlapping psychometric features and strong cross‐correlations. Due to the large and heterogeneous sample of this study, these results can serve as reference values for future studies.

## Introduction

Myotonic dystrophy type 1 (DM1) characterizes as a slow and progressive condition with marked multisystem variability. It is the second most common form of inherited muscular dystrophy and the most common among adults.^1^, ^2^, ^3^ An expansion of CTG‐repeats in the DNA is the cause behind it and directly correlates to age of onset and disease severity.^4^ DM1 typically presents with muscle wasting and weakness combined with “myotonia” and additional symptoms that culminate in impaired performance in tasks of everyday life.^5^, ^6^ Potential treatments have emerged in the last decade requiring the establishment of the best methods to measure disease progression and therapeutic impact.^7^, ^8^, ^9^ However, due to the nature of the disease and its heterogeneous phenotypes understanding and monitoring clinical progression has been a challenging task.^9^, ^10^

Differences between adult‐onset and late‐onset phenotypes have been reported before.^11^, ^12^, ^13^, ^14^, ^15^, ^16^ In addition, functional performance can differ due to age, gender, and body composition as reported not only in DM1 but also in healthy populations and other neurological disorder.^17^, ^18^, ^19^, ^20^, ^21^ This should be considered when designing clinical trials in DM1.

### Outcome measures for myotonic dystrophy

The international outcome measures for myotonic dystrophy (OMMYD) project was launched in 2011 with the aim of selecting the best available outcome measures to be used in research and clinical trials in DM1.^22^, ^23^ In the case of the functional capacity outcome measures (FCOM), the first step consisted of reviewing existing tests that could assess disease domains related to functional capacity. This was accomplished through a systematic literature review of tools previously used in DM1 or other diseases with similar characteristics.^22^ A second meeting 2 years later refined the previously selected outcomes based on three criteria: feasibility, validity, and discrimination (i.e., sensitivity and specificity to discriminate disease stages I–III and IV–V of the Muscular Impairment Rating Scale – MIRS).^23^, ^24^ The third and final meeting resulted in consensus of the FCOM tests and recommended procedures to follow when implementing these in DM1 trials.^25^

The battery of FCOM tests include: (1) 6‐minute walk test (6MWT), (2) timed 10 m walk test (10mWT) (i.e., walking at comfortable speed) and timed 10 m walk/run test (10mW/RT) (i.e., walking/running at maximum speed); (3) 30 seconds sit and stand test (30SSS), and (4) nine‐hole peg test (9HPT).^22^, ^23^, ^25^ Prior to this project, the 6MWT, 10mWT at comfortable speed, the10mWT at maximum speed and the 9HPT have undergone more rigorous testing assessing feasibility and/or reliability in adults with DM1.^26^, ^27^, ^28^ Longitudinal data in relatively small samples have been published for the 6MWT^29^ and the 10mWT at maximum speed.^13^ These findings combined highlighted the need for a large‐scale study implementing standardized procedures that would facilitate generalizability of results and to provide evidence‐based guidelines to improve reliability.

As part of the PHENODM1 natural history study and following up from the OMMYD work, we investigated FCOM tests more thoroughly and in a much larger patient sample. The aim of this cross‐sectional (*n* = 213) and longitudinal (*n* = 98) study was to explore the use of outcome measures that assess functional capacity in adults with DM1 that maybe suitable for use in clinical trials. We aimed to analyze the following: (1) feasibility and best practice for clinical trials; (2) intra‐session reliability; (3) validity, that is, association between FCOM tests and measures of muscle strength, disease severity (i.e., MIRS and Scale for Assessment and Rating of Ataxia [SARA]^30^), and, patients’ perceived functional performance; and (4) describe changes in the cohort after 1 year of natural disease progression as assessed by these FCOM. In addition, as part of the initial analysis, we aimed to investigate the degree in which demographic characteristics may affect the performance of the FCOM tests.

## Methods

### Study design

This study forms part of the observational natural history PHENODM1 study (ClinicalTrials.gov Identifier: NCT02831504). PHENODM1 is a multicenter study (i.e., The Newcastle upon Tyne Hospitals NHS Foundation Trust, and University College Hospitals NHS Foundation Trust in London) aiming to deeply phenotype an adult DM1 population to support the design of future clinical trials. This research was approved by The Newcastle and North Tyneside Ethics committee (Reference: NE/15/0178).

### Sample

A sample of 213 patients were recruited in both sites following a non‐probability strategy between October 2015 and February 2017 and included for baseline cross‐sectional analysis. The inclusion criteria were: a genetically confirmed diagnosis of DM1; ≥18 years; ability to provide informed consent and walk independently (assistive devices and orthotics allowed) for at least 10 m. The cohort recruited at one of the study sites (Newcastle) was followed up for 1 year as part of the longitudinal analysis. Patients were classified as late onset if they met two of the three following criteria: (1) first symptoms reported at the age of ≥40 years; (2) ≤200 CTG repeats; and (3) a MIRS score of I or II; otherwise they were categorized as adult (classic) phenotype.^15^, ^16^, ^31^ Participants with an early adult (juvenile) phenotype (i.e., first symptoms reported before 20 years old) were included in the adult phenotype group. There was a low possibility for congenital phenotypes (i.e., cognitively more severely impaired) to be recruited as participants should be competent to provide informed consent and considered suitable to complete all study questionnaires.

### Procedures

This study focused on the exploration of the OMMYD FCOM tests: (1) 6MWT; (2) 10mWT; referring walking at comfortable speed (i.e., patient’s selected pace); (3) 10mW/RT, referring to walking at the maximum possible and safe speed, allowing running if possible; (4) 30SSS; and (5) 9HPT.^22^, ^23^, ^25^ The following outcomes were considered for cross comparisons at baseline: (1) demographic characteristics (i.e., age, sex, age since disease onset, CTG mode at baseline, height, and weight); (2) muscle strength and capacity including: quantitative muscle testing (QMT) of ankle dorsiflexion, knee extension, and hip flexion following standardized procedures and using Microfet2 and including the best out of three attempts for analysis^15^; (3) the MIRS which is a five‐categories classification method for assessing disease progression as measured by muscle weakness manifestations and manual muscle testing^24^; (4) SARA scale which assess movement co‐ordination and has been reported as possible assessment of disease severity in DM1 regardless of the presence of ataxia or not^30^; and (5) disease‐specific patient‐reported outcome measures (PROM) that assess perceived functional performance which included the DM1‐Activ^C^ Rasch built scale and the Myotonic Dystrophy Health Index (MDHI) subscales of ability to perform activities and mobility.^32^, ^33^, ^34^, ^35^

Functional tests were assessed in a pre‐specified sequential order: (1st) 6MWT (only one trial); (2nd) 30SSS; (3rd) 10mWT; and (4th) 10mW/RT. These last three tests were requested three times (i.e., trials) as considered possible. Time for recovery in sitting position was allowed between tests. Results are reported as an average and best (i.e., fastest or highest) score. The 9HPT was performed twice per hand side. For the purposes of this manuscript, scores from the dominant side were selected. The methodology followed when testing these FCOM tests has been published as part of the OMMYD‐3 report.^25^ The10mWT and 10mW/RT standard operation procedures developed for this study differed from the ones established in the OMMYD‐3 report allowing a 1‐m flying start before initiating the stopwatch.

### Statistical analysis

IBM SPSS Statistics version 24 and R version 3.5.0 were used for all the presented analysis. Statistical analyses are outlined in depth in Data S1.

## Results

Two hundred and thirteen (*n* = 213) participants were screened between both sites with a similar distribution of men and women. Thirteen percent of the participants reported wheelchair use in daily life and 172 were classified as “adult phenotype,” The majority of our sample (81%) presented a MIRS score between II and IV, and the most commonly reported limitation to perform at least one of the FCOM tests was “poor neuromuscular control” (including issues like: impaired balance, muscle weakness, or movement disorders). Baseline demographics and clinical characteristics are summarized in Table 1.

**Table 1:** Sample demographics presenting mean and standard deviation (SD) or number (*n*) and percentage (%), and subgroups comparison.

	All	Male	Female	Significant between groups
*N*	213	104	109	
	Mean ± SD	Mean ± SD	Mean ± SD	
Age (years)	45.2 ± 14.5	47.2 ± 14.5)	43.2 ± 13.9	0.05
Height (m)	1.7 ± 0.1	1.8 ± 0.08	1.6 ± 0.07	<0.001
BMI	26.2 ± 5.9	25.9 ± 5.6	26.4 ± 6.3	0.06
Years with active education	14.9 ± 3.1	15.4 ± 3.5	14.3 ± 2.6	0.03
Years since first recalled symptoms	19.8 ± 13.6	18.1 ± 15.3	21.2 ± 12	ns
Phenotype	*n *(%)	*n *(%)	*n *(%)	
Late onset	41 (19)	24 (23)	17 (16)	ns
MIRS				0.06
I: no muscular impairment	22 (10)	15 (14)	7 (6)	
II: minimal signs of muscular impairment	59 (28)	23 (22)	36 (33)	
III: distal weakness	46 (22)	26 (25)	20 (18)	
IV: mild proximal weakness	70 (32)	30 (28)	40 (36)	
V: severe proximal weakness	16 (8)	10 (10)	6 (6)	
Walking accessories				ns
Missing data	3 (1)	0	3 (3)	
None	178 (84)	87 (84)	91 (83)	
Cane	27 (13)	14 (13)	13 (12)	
Crutches	3 (1)	2 (2)	1 (1)	
Walker	2 (2)	1 (1)	1 (1)	
Reported wheelchair use in daily life	27 (13)	11 (11)	16 (15)	ns
Reported capability to run				ns
Missing data	4 (2)	1 (1)	3 (3)	
Not possible	79 (37)	40 (38)	39 (36)	
Possible with difficulty	56 (26)	24 (23)	32 (29)	
Possible with no difficulty	74 (65)	39 (38)	35 (32)	

ns, not significant (i.e., >0.05). BMI, body mass index; MIRS, muscular impairment rating scale.

**Figure 1:**
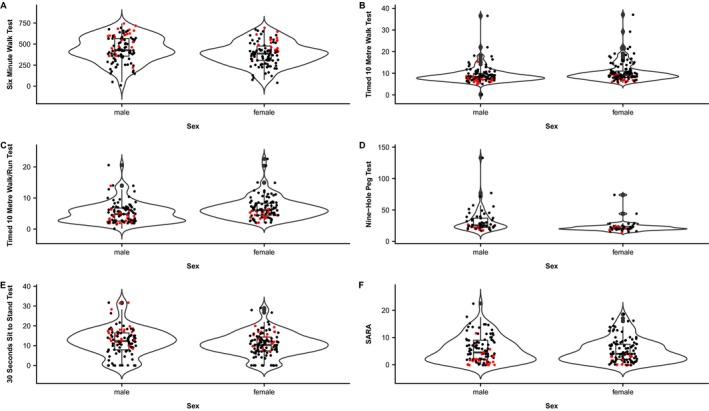
Violin plots. This figure represents the results obtained for each functional capactity outcome measure (A‐E) and the SARA (F) stratified by sex and identified by disease phenotype. These violin plots are a combination of a box plot (median, interquartile range, and adjacent values) and a density of data distribution plot. Black color represents the distribution of results corresponding to the adult phenotype and red color represents results corresponding to the late‐onset phenotype.

Figure 1 presents violin plots for each of the FCOM tests stratified by sex and differentiated based on disease phenotype (i.e., adult and late onset). Statistically significant differences between genders were identified for all assessments except for SARA score (*P* = 0.42) and 30SSS (*P* = 0.15). Body mass index (BMI) demonstrated significant impact on FCOM tests scores when incorporated into multivariate regression models, with gender also significantly influencing 9HPT. MIRS was also a highly significant factor across all FCOM tests scores. When MIRS was excluded from the model, disease phenotype was significant for all tests scores. Replacing MIRS with CTG‐repeat count in the model showed significance in three FCOM tests (6MWT, SARA, and 30SSS) but also resulted in a loss of significance of BMI and phenotype group. Age was not a significant variable influencing between subgroup differences.

Table S1 provides a full description of results classified by MIRS score and disease phenotype. Overall, MIRS classification showed a decline in performance from better scores (i.e., median) presented in the higher ranking of the MIRS (i.e., I and II) to worse in the lower ranks of the MIRS. This was observed in all tested outcomes. Phenotype subgroups differed between each other significantly in all FCOM scores, with the exception of the 9HPT in the female subgroup (Table S1).

### Feasibility and best practice

**Figure 2:**
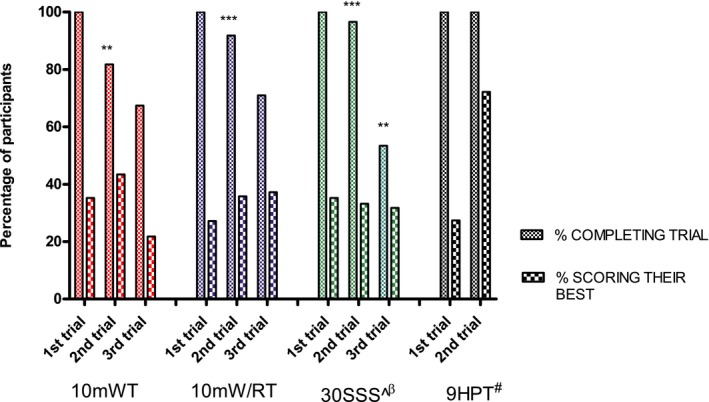
Trial completion and performance chart. This bar chart presents the percentage of participants completing each trial and the percentage performing their best at each trial (from those completing the test). **Average scores from test to test changed with a significance <0.01 (paired *t*‐test). ***Average scores from test to test changed with a significance <0.001 (paired *t*‐test). If two trials scored equally, the first trial was consider as the best trial. ^20 patients (9%) were not able to perform the 30SSS test so their best and only score considered was “zero times.” ^β^31% of the whole sample completed at least two trials with the same score (times). ^#^13% of the whole sample completed both trials with the same score (seconds).

The percentage (%) of participants completing each trial per FCOM tests (10mWT, 10mWT/RT, 30SSS, and 9HPT) and the trial in which they scored their best are presented in Figure 2. More than 80% of the participants performed at least a second trial and over 50% completed three trials in those tests required. Over 60% of the participants performed their best on their first or second trial. With the exception of the 10mWT, the most common reason (≈80%) not to carry out a second or third trial was fatigue followed by fear of falling from either the examiners’ or participants’ point of view. In the case of the 10mWT the most common reason not to repeat was consistency between the first and second trials as judged by the assessor. In the case of the 30SSS and the 10mW/RT participants with a milder presentation of the disease (i.e., MIRS I and II) scored better at the second or third trial. The majority (62%) of participants with a more severe presentation (i.e., MIRS V) scored their best at the first attempt. Twenty patients (9%) were not able to perform the 30SSS test without support so their best and only score considered was recorded as “zero times.” Three falls were reported for the 6MWT and two for the 10mW/RT although only one of these resulted in stopping the test (i.e., 6MWT) with no resultant injuries.

### Intra‐session reliability

All FCOM tests performed more than once showed high intraclass correlation coefficients (ICC_2,1_): 10mWT (ICC = 0.99, 95% confidence interval [CI] 0.99–0.99); 10mW/RT (ICC = 0.99, 95% CI 0.98–0.99); 30SSS (ICC = 0.96, 95% CI 0.89–0.98); and 9HPT (ICC = 0.90, 95% CI 0.83–9.94). There was a statistically significant difference (*P* < 0.001) from the first trial to the second on all FCOM tests and between the second and third trials only for the 30SSS (Fig. 2). Bland‐Altman plots between the second and the third trials of the 10mWT and 10mW/RT confirmed an absolute agreement between these trials (mean difference of 0.5 sec and a 95% limit of agreement between −2.0 and 2.1 sec). There was a significant difference (*P* = 0.004) between the 9HPT completed with the dominant side and the non‐dominant side of 1.2 sec (standard deviation [SD] 6.7 sec) (Table S1).

### Construct validity

There were significant correlations between most FCOM tests (average scores), and measures of muscle strength (QMT), SARA score, and the PROMs results (Table 2). Less than moderate correlations (i.e., correlation coefficient <0.5) were mainly found between the FCOM tests and measures of muscle strength, whereas correlations with the SARA were all in the moderate range (i.e., correlation coefficients ≥0.5) and correlations with the PROMs were all in the moderate to strong range (i.e., between 0.5 and 0.9) except for the 9HPT (i.e., <0.5). The 30SSS test showed a significant correlation with MDHI‐fatigue subscale, which is not presented in this table (*r* = 0.5, *P* < 0.01). There was a strong correlation (*r* = 0.8, *P* < 0.01) identified between the walking capacity tests (i.e., 6MWT, 10mWT, and 10mW/RT) (data not presented in table).

**Table 2:** Correlation scores between outcome measures.^a^

Outcome measure	SARA	Knee extensors QMT	Hip flexors QMT	Ankle dorsi‐flexors QMT	MDHI ‐ability to perform activities subscale	MDHI ‐mobility subscale	DM1‐ActivC TOTAL score
6MWT	(−)0.65	0.47	0.51	0.45	(−)0.64	(−)0.73	0.69
10mWT	0.65	(−)0.36	(−)0.45	(−)0.43	0.63	0.73	(−)0.67
10mW/RT	0.55	(−)0.32	(−)0.51	(−)0.47	0.58	0.66	(−)0.59
30SSS	(−)0.67	0.44	0.53	0.52	(−)0.58	(−)0.65	0.65
9HPT	0.55	ns	ns	(−)0.26	0.23	0.32	(−)0.41

Outcome measure: 6MWT (6‐minute walk test), 10mWT (timed 10 m walk test), 10mW/RT (timed 10 m walk/run test), 30SSS (30 sec sit and stand test), and 9HPT (nine‐hole peg test). SARA, scale for assessment and rating of ataxia; QMT, quantitative muscle testing (best score of three); MDHI, Myotonic Dystrophy Health Index; DM1, myotonic dystrophy type 1.

a All correlations presented showed to be significant at the <0.01 level (two tailed).

### Longitudinal analysis

**Figure 3:**
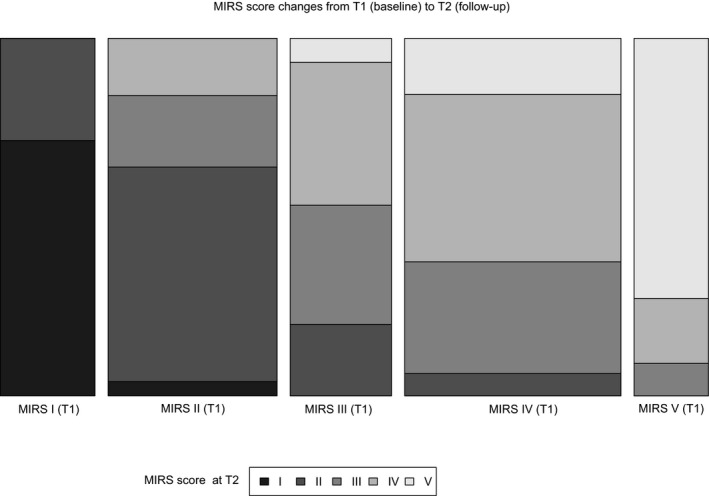
Muscular Impairment Rating Scale (MIRS) changes from baseline (T1) to follow‐up (T2). This bar chart presents the whole sample classified based on the MIRS score assigned at baseline (T1) and differentiated in gray scale based on their MIRS score assigned at follow‐up (T2). The width of each column has been defined based on the amount of patients on each T1‐MIRS group.

From 110 patients screened in Newcastle, 98 completed a second follow‐up visit 12 months apart. From the 12 losses in follow‐up, 10 were study dropouts or failures to attend within visit window and two were due to serious events not related to the study. Demographics at baseline and the mean and SD of those patients’ scores at baseline (T1) and follow‐up visit (T2) are presented in Table 3 as a whole sample and in Table 4 divided by disease‐phenotype subgroups. There was a clear distinction in disease phenotype between adult and late‐onset subgroups with all parameters showing a statistically significant difference at baseline (T1). With the exception of the 9HPT, all FCOM tests showed statistical significant changes over time, as did SARA. This significant decline was not detected with muscle strength assessments, nor with QMT nor MIRS (Fig. 3). Both phenotype subgroups showed similar and significant changes over time. The adult phenotype subgroup showed a statistically significant change (of improvement) in muscle strength scores which was not observed in the late‐onset subgroup. Patients that scored a MIRS of III and IV at baseline also showed variability (of improvement) at follow‐up (Fig. 3).

**Table 3:** Longitudinal (12 months) data.

	All (adult and late‐onset phenotype)				
	(T1)	(T2)			
	Mean (SD)	Mean change	Percentage	Lower CI to upper CI	Level of significance
Demographics					
*N*=	98				
Females (*n*)	43 (44%)				
Age (years)	46 (14)				
Height (m)	2 (6)				
BMI	26 (6)				
Late‐onset phenotype (*n*)	22 (23%)				
Progenitor allele (CTG count)	256 (184)				
CTG mode (CTG count)	500 (357)	3.8	1%	7.4–15	ns
Outcome measures					
6MWT (m)	425 (94)	−35.3	−8%	−21.1 to −49.6	<0.001
10mWT (sec) – average	9.6 (4.3)	1.1	11%	1.8–0.3	0.004
10mWT (sec) – best	9.2 (4.2)	1.2	13%	1.9–0.4	0.002
10mW/RT (sec) – average	5.3 (3.0)	0.8	15%	1.3–0.3	0.003
10mW/RT (sec) – best	5.0 (3.0)	0.9	18%	1.5–0.3	0.002
30SSS (times) – average	11.2 (5.8)	−0.7	−6%	−0.1 to −1.3	0.03
30SSS (times) – best	12.1 (6.3)	−1.2	−10%	−0.5 to −1.8	0.001
9HPT (sec) – average	27.7 (13.7)	0.7	3%	2.9 to −1.4	ns
9HPT (sec) – best	25.7 (12.4)	0.9	4%	3.1 to −1.2	ns
SARA (score)	6.1 (4.9)	1.1	18%	1.7–0.5	0.001
Knee extensors QMT (lb)	46.4 (19.4)	2.1	5%	5.4 to −1.1	ns
Hip flexors QMT (lb)	33.7 (13.1)	2.2	6%	4.8 to −0.5	ns
Ankle dorsi‐flexors QMT (lb)	25.6 (13.3)	1.9	7%	4.5 to −0.7	ns

Results at baseline (T1) are presented as mean and standard deviation (SD) and changes over time are presented (T2) as mean change and 95% confidence intervals (CI). Outcome Measure: 6MWT (6‐minute walk test), 10mWT (timed 10 m walk test), 10mW/RT (timed 10 m walk/run test), 30SSS (30 sec sit and stand test), 9HPT (nine‐hole peg test), and SARA (scale for assessment and rating of ataxia). BMI, body mass index; QMT, quantitative muscle testing (best score of three).

**Table 4:** Longitudinal (12 months) data.

	Adult phenotype						Late‐onset phenotype					
	(T1)		(T2)				(T1)		(T2)			
	Mean (SD)		Mean change	Percentage	Lower CI to upper CI	Level of significance	Mean (SD)		Mean change	Percentage	Lower CI to upper CI	Level of significance
Demographics												
*N* =	76						22					
Females (*n*)	33 (43%)						10 (45%)					
Age (years)	43 (13)						57 (13)					
Height (m)	2 (10)						2 (9)					
BMI	26 (6)						25 (4)					
Outcome measures												
6MWT (m)	381 (153)		−36.2	−10%	(−19.3 to −53.1)	<0.001	588 (94)		−32.0	−5%	(−5.8 to −58.2)	0.02
10mWT (sec) – average	10.3 (4.6)		1.3	12%	(2.2–0.3)	0.009	7.2 (1.3)		0.4	6%	(0.8–0.0)	0.04
10mWT (sec) – best	9.9 (4.5)		1.4	14%	(2.3–0.4)	0.004	6.9 (1.3)		0.4	6%	(0.8–0.1)	0.03
10mW/RT (sec) – average	6.0 (3.2)		0.9	14%	(1.5–0.2)	0.01	3.3 (1.3)		0.6	18%	(1.1–0.1)	0.02
10mW/RT (sec) – best	5.6 (3.1)		0.9	17%	(1.6–0.2)	0.01	2.9 (1.3)		0.8	28%	(1.3–0.3)	0.004
30SSS (times) – average	10.1 (5.4)		−0.6	−6%	(0.2 to −1.3)	ns	15 (5.7)		−1.1	−7%	(−0.2 to −2.0)	0.02
30SSS (times) – best	11 (5.8)		−1.0	−10%	(−0.2 to −1.8)	0.01	16.1 (5.8)		−1.6	−10%	(−0.6 to −2.5)	0.002
9HPT (sec) – average	29.6 (15)		1.0	3%	(3.8 to −1.7)	ns	21.3 (2.7)		−0.2	−1%	(0.9 to −1.3)	ns
9HPT (sec) – best	27.1 (14)		1.4	5%	(4.2 to −1.4)	ns	20.7 (2.7)		−0.6	−3%	(0.6 to −1.9)	ns
SARA (score)	7.5 (4.8)		1.1	15%	(1.9–0.3)	0.01	2 (4.9)		1.1	55%	(2.1–0.1)	0.04
Knee extensors QMT (lb)	44.2 (19)		1.1	2%	(4.4 to −2.2)	ns	54.3 (19.1)		5.8	11%	(15.6 to −4.0)	ns
Hip flexors QMT (lb)	31.6 (14)		3.3	10%	(6.3–0.3)	0.03	37.3 (12)		−1.9	−5%	(4.0 to −7.7)	ns
Ankle dorsi‐flexors QMT (lb)	21.6 (12)		3.5	16%	(6.3–0.6)	0.02	33.3 (15.4)		−2.2	−7%	(3.3 to −7.7)	ns

Results at baseline (T1) are presented as mean and standard deviation (SD) and changes over time are presented (T2) as mean change and 95% confidence intervals (CI). Outcome Measure: 6MWT (6‐minute walk test), 10mWT (timed 10 m walk test), 10mW/RT (timed 10 m walk/run test), 30SSS (30 sec sit and stand test), 9HPT (nine‐hole peg test), SARA (scale for assessment and rating of ataxia), and QMT: quantitative muscle testing (best score of three). BMI, body mass index.

## Discussion

Assessing functional capacity in people with DM1 is essential to monitor natural disease progression and the possible effect of any intervention. This study explores feasibility, intra‐session reliability, validity, and sensitivity to detect change of the OMMYD suggested FCOM tests for the first time after the establishment of the OMMYD FCOM guidelines.^25^

Significant differences in performance scores were observed between genders (due to body composition differences and muscle strength) with men commonly scoring higher than women. In DM1, however, it has been reported that men more frequently have muscular weakness and disability which at first glance would not be reflected on the overall scores of these FCOM tests.^11^ However, when comparing the differences between subgroups and controlling for variables expected to impact on these scores (i.e., age, height, BMI, MIRS and CTG‐repeats mode at baseline), the significance between subgroups comparison changed (Table S1). For example, after performing this model, walking tests (i.e., 6MWT and 10mWT) significance between male and female disappeared and the difference in 30SSS became significant highlighting the relevant influence of BMI and MIRS in these scores. The late‐onset phenotype subgroup differed significantly from the adult phenotype in all outcomes, which proves once more that generalizability of results should be cautious when considering data from mixed‐phenotypic samples and the association with muscular strength as measured by MIRS.^11^, ^12^, ^13^, ^14^, ^15^, ^16^ However, using five categories of disease severity (i.e., MIRS) as compared to only two (i.e., Phenotype) was shown to be more strongly associated with patient performance. SARA scores were not influenced by age, sex or height, making it a suitable outcome for wider comparisons.

Mean values obtained on these FCOM tests are comparable to other relevant adult neurological conditions.^36^, ^37^ Normative data for 30SSS for an adult establishes scores from 13 to 15 full stands (i.e., repetitions) for women and from 14 to 17 full stands for men.^38^, ^39^ In our population only participants completing a third trial of the test accomplished these scores and these participants were generally the least affected (i.e., MIRS I, II, and III). In fact, the average score obtained as a group on the first trial was below the cut‐off value predicted for a population between 60 and 70 years old.^39^, ^40^

We identified that when assessing functional outcomes (i.e., 10mWT, 6MWT, timed‐stands test, and the timed up‐and‐go test) in DM1, at least half of the participants performed their best test at either the second or the third trial.^41^ Based on the resulting intra‐session ICC_2,1_ and the non‐difference detected between the second and the third trial observed for the 10mWT and the 10mW/RT we suggest that two trials of these tests will be sufficient to provide a valid and reliable score. Still, due to the significant change from the first to the second trials, there seems to be a learning effect that should be considered. In addition, it is feasible to perform these tests at least once all together as 96% of the participants completed all five FCOM tests, 6MWT inclusive. However, as expected, not all participants were able to complete all assessments three times. Fear of falling was the most common reason for failure to complete (Fig. 2). The 30SSS, had the lowest compliance rate; however, the more trials that were completed, the greater chance participants had to perform their best trial. This improvement was seen for participants with MIRS I to III. In this test, the reduced compliance was mostly attributed to fatigue which concurs with what has been suggested before for this test^39^, ^42^ but also correlated with the MDHI‐fatigue score (*r* = 0.5, *P* < 0.01). Due to disease‐associated limiting factors such as fatigue, pain, and poor balance, an exhaustive examination of these patients is discouraged and a careful consideration to reduce the number of assessments or visit length is recommended. The 10mWT and 10mW/RT are tests relatively short in time, making them more feasible for trial repetition and when compared to the 6MWT; all three assess walking capacity and demonstrated strong correlation between each other. Due to the observed variability from trial to trial, using the “best” trial for analysis would risk ignoring the natural variation of the test, whereas the average of repeated trials may be more representative of a true score.^43^

Our results provide evidence of the association of these FCOM tests with muscle strength and the SARA assessments as surrogates of disease severity. Twenty‐five percent of the performance to walk and to stand up from a chair can be explained by muscle strength.^13^, ^26^, ^27^, ^44^, ^45^ The 6MWT and 10mWT maintain similar correlation trends among all tests. Once more, knee extensors and ankle dorsiflexors strength have shown significant impact on test performance.^13^, ^27^ The minimal correlation identified between 9HPT and ankle dorsiflexion has not been considered relevant assuming this as a spurious finding.

With the exception of 9HPT, all FCOM tests and SARA showed a statistically significant change after 1 year in this large and heterogeneous study population. Still, the clinical significance and impact on disease burden of these changes needs further investigation. QMT muscle testing and MIRS scores did not show an overall significant change but even gave hints of improvement. When assessing the adult phenotype subgroup independently, some QMT scores showed significant improvement from baseline (T1) to follow‐up (T2) (Table 4); and some participants classified with a MIRS IV at T1 escalated up to a MIRS II at T2 (Fig. 3). Even though this is a progressive disease and it would be unlikely to detect improvement when there has been no intervention, we cannot accept or reject these findings as multiple factors could influence on muscle strength scoring from T1 to T2. Other studies that have investigated natural disease progression in DM1 over a longer period of time,^16^, ^31^ identified differences in speed and magnitude of disease progression between the late onset and the adult phenotype. These differences were not detected at our 1‐year study.

This study has several limitations. First, all assessments tested in this study have been completed on a 1‐day visit. Having two independent visits closer in time (1 day or 1 week apart) or having two independent assessors repeating the examinations would have inform conclusions regarding validity and standard error of measurement. Secondly, this study does not address other factors that could influence in performance such as: physical activity levels, myotonia, fatigability, and co‐morbidities. Additionally, this study has been completed at two different sites involving seven trained assessors (three of which over 80% of the assessments), which may impact on the variability observed in the muscle strength scores (including MIRS). Lastly, even though the overall sample is one of the largest studied to date in DM1, a rare disease, it, did not have sufficient statistical power to allow for subgroup comparisons.

Based on the lessons learned from this study, the authors have highlighted the points below for consideration when including any of these tests as outcomes in clinical trials or research studies in DM1:

- Follow OMMYD methodological references when selecting an outcome and methodology suitable for DM1 adults.^25^ *Variants from these guidelines applied in this study: flying start allowed for the 10mWT and the 10mW/RT.
- Correct for BMI and disease severity (i.e., MIRS) when comparing groups.
- Stratify your sample based on disease phenotype and sex if possible.
- Perform at least two trials for any of the selected FCOM to prevent bias from learning effect and include the best of these for your analysis.
- Although not capturing walking endurance as the 6MWT, the 10mWT, and 10mW/RT reflect similar functional walking capacity making them feasible alternatives to implement in clinical trials allowing trial repetition and the need of relatively shorter testing‐time and space.
- A repetitive 30SSS test may add information about other disease symptoms impacting on test performance such as balance and fatigue.

## Conclusion

This study reports the first full exploration of five FCOM tests (6MWT, 10mWT, the 10mW/RT, the 30SSS, and the 9HPT) in adults with genetically and phenotypically determined DM1 as recommended by the OMMYD group.^46^ The large sample size and the standardized methodology followed allow these results to be considered as appropriate reference values for future clinical trials. Overall, this study has defined suitable methodology for future studies assessing interventions offering improvement in DM1 patients’ functional capacity.

## Conflict of Interest

Professor Lochmüller reports consultancy and financial support for research projects and clinical trials by AMO Pharma, Biogen, Desitin, GW Pharma, Pfizer, PTC Therapeutics, Roche, Santhera, Sarepta, Satellos, and Ultragenyx. He is the Editor‐in‐chief for the Journal of Neuromuscular Disease (IOS Press).

## Supporting information


**Data S1.** Statistical analysis plan and description.

**Table S1.** Table with FCOM results (i.e., median and quartiles, and, mean and standard deviation) stratified according to parameters of disease severity (i.e., MIRS score and disease‐phenotype subgroup).
