# Supplementary material for: Analysis of the functional capacity outcome measures for myotonic dystrophy
Source: Ann Clin Transl Neurol. 2019 Jul 22;6(8):1487–97. doi: 10.1002/acn3.50845 (PMC6689676; doi:10.1002/acn3.50845)
Supplement: Supplementary file 1 — Data S1. Statistical analysis plan and description. [file ACN3-6-1487-s001.docx]

Statistical analysis

Normality was determined using the Shapiro-Wilk test. Descriptive statistics were used to report participants’ demographic and clinical characteristics at baseline and the results of outcomes analysed. Distribution of scores between subgroups (i.e. sex and disease phenotype) was compared using Independent samples t-test and Mann-Whitney U-test or Pearson Chi Squared for categorical data. Sex, age, height, BMI (weight (kg) / [height (m)^2^]), disease phenotype were all considered potential predictors of walking performance and so were included as covariates within all subsequent regression modelling, with any significance noted in the results section. Models were then ran to compare the effect of including either or both MIRS and CTG-repeats count alongside the other covariates. Additionally, FCOM tests results were compared between subgroups by categories based on their disease severity score (i.e. MIRS and disease phenotype) and sex.

Due to sample size limitations, a linear model was only performed when comparing subgroups as a whole sample and not when compared by categories. Intraclass correlation coefficients (ICC_2,1_) were used for analyses of intra-session (i.e. between trial to trial) reliability (27, 28). Paired t-test between trials 1 and 2 and then 2 and 3 were performed to measure differences in scores from trial to trial and as an insight into a possible learning effect of the test. Whenever two trials showed no significant differences between scores, Bland-Altman plots were created to identify the mean difference and 95% limits of agreement as confirmation of absolute reliability (29). To assess the construct validity of the FCOM tests, Spearman rank correlation analyses was performed examining the relationship between FCOM tests and muscle strength, SARA and PROM. Correlation coefficients ≥0.5 were considered moderate and >0.7 as strong (30). For longitudinal analysis, paired-sample t-tests were performed for within group and subgroup comparisons. A univariate model correcting for baseline scores was performed to compare the degree of change from one time point to another between subgroups (i.e. disease phenotype). Statistical significance was always established as *p* <0.05 although when considered appropriate non-significant values were also presented for reference.

Lead statistician: Dr. Alasdair Blain, Institute of Neurosciences, Newcastle University, UK.
