# Supplementary material for: Analysis of the functional capacity outcome measures for myotonic dystrophy
Source: Ann Clin Transl Neurol. 2019 Jul 22;6(8):1487–97. doi: 10.1002/acn3.50845 (PMC6689676; doi:10.1002/acn3.50845)
Supplement: Supplementary file 2 — Table S1. Table with FCOM results (i.e., median and quartiles, and, mean and standard deviation) stratified according to parameters of disease severity (i.e., MIRS score and disease‐phenotype subgroup). [file ACN3-6-1487-s002.docx]

| **Supplementary Table:** This table presents the distribution of FCOM test results stratified according to parameters of disease severity (i.e. MIRS score and disease-phenotype subgroup) | | | | | | | | | | | | | | | | | | | | | | | | | | | | | | | | | | | | |
| --- | --- | --- | --- | --- | --- | --- | --- | --- | --- | --- | --- | --- | --- | --- | --- | --- | --- | --- | --- | --- | --- | --- | --- | --- | --- | --- | --- | --- | --- | --- | --- | --- | --- | --- | --- | --- |
|  | **All (male + female)** | | | | | | | | | | | **Male subgroup** | | | | | | | | | | **Female subgroup** | | | | | | | | | | | | *Level of significance* | | |
| **Outcome Measure** | **n** | **% female** | **Median** | | **25%** | | **75%** | | **mean** | | **SD** | **Median** | | **25%** | | **75%** | | **mean** | | **SD** | | **Median** | | **25%** | | **75%** | | | **mean** | | **SD** | | | *between sex* | *correcting to height, BMI and MIRS* | |
| **6MWT (m)** | **206** |  | **409** | | **317** | | **537** | | **414** | | **150** | **427** | | **354** | | **566** | | **440** | | **155** | | **387** | | **3** | | **485** | | | **387** | | **140** | | | 0.01 | 0.5 | |
| MIRS I | 21 | 29% | 595 | | 528 | | 646 | | 562 | | 110 | 595 | | 528 | | 609 | | 562 | | 109 | | 598 | | 425 | | 650 | | | 562 | | 123 | | | *ns* |  | |
| MIRS II | 59 | 59% | 525 | | 416 | | 574 | | 497 | | 132 | 564 | | 475 | | 630 | | 539 | | 131 | | 490 | | 412 | | 557 | | | 469 | | 126 | | | 0.04 |  | |
| MIRS III | 45 | 42% | 390 | | 345 | | 434 | | 385 | | 111 | 412 | | 379 | | 464 | | 414 | | 117 | | 358 | | 251 | | 408 | | | 345 | | 89 | | | 0.04 |  | |
| MIRS IV | 66 | 56% | 352 | | 275 | | 424 | | 354 | | 121 | 384 | | 315 | | 456 | | 395 | | 120 | | 312 | | 252 | | 407 | | | 322 | | 112 | | | 0.01 |  | |
| MIRS V | 15 | 33% | 256 | | 150 | | 360 | | 238 | | 138 | 216 | | 150 | | 360 | | 229 | | 145 | | 312 | | 225 | | 322 | | | 257 | | 134 | | | *ns* |  | |
| **adult onset phenotype** | **166** | 52% | **387** | | **294** | | **482** | | **385** | | **143** | **403** | | **315** | | **525** | | **408** | | **149** | | **364** | | **267** | | **427** | | | **364** | | **134** | | | 0.04 |  | |
| **late-onset phenotype** | **40** | 40% | **548** | | **450** | | **613** | | **532***** | | **117** | **590** | | **453** | | **623** | | **544***** | | **130** | | **528** | | **436** | | **570** | | | **514***** | | **96** | | | *ns* |  | |
|  |  |  |  | |  | |  | |  | |  |  | |  | |  | |  | |  | |  | |  | |  | | |  | |  | | |  |  | |
| **10mWT (s) - average** | **213** |  | **9** | | **9** | | **11** | | **10** | | **4** | **8** | | **7** | | **10** | | **9** | | **4** | | **9** | | **8** | | **11** | | | **11** | | **5** | | | 0.02 | 0.10 | |
| MIRS I | 22 | 32% | 7 | | 6 | | 8 | | 7 | | 1 | 7 | | 6 | | 8 | | 7 | | 1 | | 6 | | 6 | | 9 | | | 7 | | 2 | | | *ns* |  | |
| MIRS II | 59 | 61% | 8 | | 7 | | 9 | | 8 | | 2 | 8 | | 6 | | 9 | | 8 | | 2 | | 8 | | 7 | | 9 | | | 8 | | 2 | | | *ns* |  | |
| MIRS III | 46 | 43% | 9 | | 8 | | 10 | | 10 | | 4 | 9 | | 7 | | 9 | | 9 | | 3 | | 9 | | 8 | | 13 | | | 11 | | 5 | | | 0.04 |  | |
| MIRS IV | 70 | 56% | 10 | | 9 | | 12 | | 11 | | 5 | 9 | | 8 | | 11 | | 10 | | 3 | | 10 | | 9 | | 14 | | | 12 | | 6 | | | *ns* |  | |
| MIRS V | 16 | 38% | 11 | | 10 | | 18 | | 13 | | 8 | 11 | | 9 | | 14 | | 13 | | 10 | | 12 | | 10 | | 18 | | | 14 | | 4 | | | *ns* |  | |
| **adult onset phenotype** | **172** | 53% | **9** | | **8** | | **11** | | **11** | | **5** | **9** | | **7** | | **10** | | **9** | | **3** | | **10** | | **8** | | **12** | | | **11** | | **5** | | | 0.04 |  | |
| **late-onset phenotype** | **41** | 41% | **7** | | **6** | | **8** | | **7***** | | **2** | **7** | | **6** | | **8** | | **7**** | | **2** | | **7** | | **6** | | **8** | | | **7***** | | **11** | | | *ns* |  | |
|  |  |  |  | |  | |  | |  | |  |  | |  | |  | |  | |  | |  | |  | |  | | |  | |  | | |  |  | |
| **10mWT (s) - best** | **213** |  | **6** | | **3** | | **8** | | **6** | | **4** | **8** | | **7** | | **10** | | **9** | | **4** | | **9** | | **8** | | **11** | | | **10** | | **4** | | | 0.03 | 0.06 | |
| MIRS I | 22 | 32% | 7 | | 6 | | 8 | | 7 | | 1 | 7 | | 6 | | 7 | | 7 | | 1 | | 6 | | 6 | | 9 | | | 7 | | 2 | | | *ns* |  | |
| MIRS II | 59 | 61% | 8 | | 6 | | 9 | | 8 | | 2 | 7 | | 6 | | 8 | | 8 | | 2 | | 8 | | 7 | | 9 | | | 8 | | 2 | | | *ns* |  | |
| MIRS III | 46 | 43% | 8 | | 8 | | 10 | | 10 | | 4 | 8 | | 7 | | 9 | | 9 | | 3 | | 9 | | 8 | | 12 | | | 11 | | 5 | | | *ns* |  | |
| MIRS IV | 70 | 56% | 10 | | 8 | | 12 | | 11 | | 4 | 9 | | 8 | | 11 | | 10 | | 3 | | 10 | | 8 | | 14 | | | 12 | | 5 | | | *ns* |  | |
| MIRS V | 16 | 38% | 11 | | 10 | | 15 | | 13 | | 8 | 11 | | 9 | | 14 | | 13 | | 10 | | 12 | | 10 | | 15 | | | 13 | | 3 | | | *ns* |  | |
| **adult onset phenotype** | **172** | 53% | **9** | | **8** | | **11** | | **9** | | **5** | **8** | | **7** | | **9** | | **9** | | **3** | | **9** | | **8** | | **12** | | | **10** | | **4** | | | 0.06 |  | |
| **late-onset phenotype** | **41** | 41% | **7** | | **6** | | **8** | | **7***** | | **2** | **7** | | **6** | | **8** | | **7**** | | **2** | | **7** | | **6** | | **8** | | | **7**** | | **1** | | | *ns* |  | |
|  |  |  |  | |  | |  | |  | |  |  | |  | |  | |  | |  | |  | |  | |  | | |  | |  | | |  |  | |
| **10mW/RT (s) - average** | **204** |  | **6** | | **3** | | **8** | | **6** | | **3** | **5** | | **3** | | **7** | | **5** | | **3** | | **6** | | **5** | | **8** | | | **7** | | **4** | | | 0.002 | 0.005 | |
| MIRS I | 22 | 32% | 3 | | 2 | | 3 | | 3 | | 1 | 3 | | 2 | | 3 | | 3 | | 1 | | 3 | | 2 | | 6 | | | 4 | | 2 | | | *ns* |  | |
| MIRS II | 59 | 61% | 5 | | 3 | | 6 | | 5 | | 3 | 3 | | 2 | | 6 | | 4 | | 3 | | 5 | | 4 | | 6 | | | 5 | | 2 | | | *ns* |  | |
| MIRS III | 44 | 45% | 6 | | 4 | | 7 | | 6 | | 3 | 5 | | 3 | | 7 | | 6 | | 4 | | 7 | | 6 | | 9 | | | 7 | | 2 | | | *ns* |  | |
| MIRS IV | 64 | 56% | 7 | | 5 | | 9 | | 7 | | 4 | 6 | | 5 | | 8 | | 7 | | 3 | | 7 | | 6 | | 10 | | | 8 | | 4 | | | *ns* |  | |
| MIRS V | 15 | 40% | 8 | | 7 | | 11 | | 9 | | 4 | 7 | | 5 | | 10 | | 7 | | 4 | | 10 | | 8 | | 12 | | | 10 | | 3 | | | *ns* |  | |
| **adult phenotype** | **163** | 56% | **6** | | **4** | | **8** | | **7** | | **4** | **5** | | **3** | | **7** | | **5** | | **4** | | **7** | | **5** | | **9** | | | **7** | | **4** | | | 0.01 |  | |
| **late-onset phenotype** | **41** | 41% | **4** | | **3** | | **5** | | **4***** | | **2** | **3** | | **2** | | **5** | | **4**** | | **3** | | **5** | | **4** | | **6** | | | **5***** | | **1** | | | *ns* |  | |
|  |  |  |  | |  | |  | |  | |  |  | |  | |  | |  | |  | |  | |  | |  | | |  | |  | | |  |  | |
| **10mW/RT (s) - best** | **204** |  | **5** | | **3** | | **7** | | **10** | | **4** | **5** | | **2** | | **7** | | **5** | | **3** | | **6** | | **4** | | **8** | | | **6** | | **3** | | | 0.002 | 0.004 | |
| MIRS I | 22 | 32% | 2 | | 2 | | 3 | | 3 | | 1 | 2 | | 2 | | 3 | | 2 | | 1 | | 3 | | 2 | | 5 | | | 3 | | 2 | | | *ns* |  | |
| MIRS II | 59 | 61% | 4 | | 3 | | 6 | | 4 | | 3 | 3 | | 2 | | 5 | | 4 | | 3 | | 5 | | 4 | | 6 | | | 5 | | 2 | | | *ns* |  | |
| MIRS III | 44 | 45% | 6 | | 4 | | 7 | | 6 | | 3 | 5 | | 3 | | 7 | | 5 | | 3 | | 6 | | 5 | | 8 | | | 7 | | 2 | | | *ns* |  | |
| MIRS IV | 64 | 56% | 7 | | 5 | | 9 | | 7 | | 4 | 6 | | 4 | | 7 | | 6 | | 3 | | 7 | | 5 | | 10 | | | 8 | | 4 | | | *ns* |  | |
| MIRS V | 15 | 40% | 8 | | 7 | | 10 | | 8 | | 4 | 7 | | 5 | | 9 | | 7 | | 4 | | 9 | | 7 | | 12 | | | 10 | | 3 | | | *ns* |  | |
| **adult onset phenotype** | **163** | 56% | **6** | | **4** | | **8** | | **6** | | **4** | **5** | | **2** | | **7** | | **5** | | **3** | | **6** | | **5** | | **9** | | | **6** | | **4** | | | 0.009 |  | |
| **late-onset phenotype** | **41** | 41% | **3** | | **2** | | **5** | | **4***** | | **2** | **3** | | **2** | | **4** | | **4**** | | **2** | | **7** | | **3** | | **6** | | | **7***** | | **1** | | | *ns* |  | |
|  |  |  |  | |  | |  | |  | |  |  | |  | |  | |  | |  | |  | |  | |  | | |  | |  | | |  |  | |
| **30SSS (times) - average** | **213** |  | **12** | | **8** | | **15** | | **11** | | **6** | **12** | | **8** | | **17** | | **12** | | **7** | | **10** | | **7** | | **14** | | | **11** | | **6** | | | 0.2 | 0.004 | |
| MIRS I | 22 | 32% | 17 | | 13 | | 19 | | 17 | | 6 | 17 | | 13 | | 20 | | 19 | | 7 | | 17 | | 9 | | 19 | | | 14 | | 5 | | | *ns* |  | |
| MIRS II | 59 | 61% | 14 | | 10 | | 18 | | 14 | | 6 | 15 | | 12 | | 18 | | 14 | | 5 | | 14 | | 10 | | 17 | | | 14 | | 6 | | | *ns* |  | |
| MIRS III | 46 | 43% | 12 | | 9 | | 15 | | 12 | | 7 | 13 | | 10 | | 15 | | 13 | | 7 | | 11 | | 9 | | 14 | | | 10 | | 6 | | | *ns* |  | |
| MIRS IV | 70 | 43% | 9 | | 7 | | 12 | | 9 | | 4 | 10 | | 7 | | 13 | | 10 | | 4 | | 9 | | 6 | | 12 | | | 8 | | 4 | | | *ns* |  | |
| MIRS V | 16 | 38% | 1 | | 0 | | 6 | | 3 | | 4 | 1 | | 0 | | 5 | | 3 | | 4 | | 3 | | 0 | | 7 | | | 4 | | 5 | | | *ns* |  | |
| **adult onset phenotype** | **172** | 53% | **11** | | **7** | | **14** | | **11** | | **6** | **13** | | **9** | | **17** | | **13** | | **7** | | **10** | | **7** | | **14** | | | **11** | | **6** | | | *ns* |  | |
| **late-onset phenotype** | **41** | 41% | **15** | | **12** | | **18** | | **15***** | | **6** | **16** | | **12** | | **18** | | **15***** | | **7** | | **15** | | **11** | | **17** | | | **14*** | | **4** | | | *ns* |  | |
|  |  |  |  | |  | |  | |  | |  |  | |  | |  | |  | |  | |  | |  | |  | | |  | |  | | |  |  | |
| **30SSS (times) - best** | **213** |  | **13** | | **8** | | **17** | | **12** | | **7** | **14** | | **8** | | **18** | | **13** | | **7** | | **11** | | **8** | | **15** | | | **12** | | **6** | | | 0.1 | 0.003 | |
| MIRS I | 22 | 32% | 18 | | 14 | | 20 | | 18 | | 7 | 18 | | 14 | | 22 | | 20 | | 7 | | 18 | | 9 | | 20 | | | 15 | | 6 | | | *ns* |  | |
| MIRS II | 59 | 61% | 15 | | 11 | | 19 | | 15 | | 6 | 17 | | 13 | | 19 | | 15 | | 6 | | 15 | | 10 | | 18 | | | 15 | | 6 | | | *ns* |  | |
| MIRS III | 46 | 43% | 14 | | 10 | | 17 | | 13 | | 7 | 16 | | 12 | | 17 | | 15 | | 8 | | 11 | | 9 | | 15 | | | 11 | | 6 | | | *ns* |  | |
| MIRS IV | 70 | 43% | 10 | | 7 | | 12 | | 10 | | 5 | 11 | | 8 | | 13 | | 10 | | 5 | | 10 | | 7 | | 12 | | | 9 | | 5 | | | *ns* |  | |
| MIRS V | 16 | 38% | 1 | | 0 | | 6 | | 4 | | 5 | 1 | | 0 | | 5 | | 3 | | 5 | | 3 | | 0 | | 7 | | | 4 | | 6 | | | *ns* |  | |
| **adult onset phenotype** | **172** | 53% | **12** | | **7** | | **16** | | **11** | | **7** | **14** | | **9** | | **18** | | **13** | | **7** | | **11** | | **8** | | **15** | | | **12** | | **6** | | | *ns* |  | |
| **late-onset phenotype** | **41** | 41% | **16** | | **13** | | **19** | | **16**** | | **6** | **16** | | **13** | | **19** | | **17***** | | **7** | | **16** | | **11** | | **18** | | | **15*** | | **4** | | | *ns* |  | |
|  |  |  |  | |  | |  | |  | |  |  | |  | |  | |  | |  | |  | |  | |  | | |  | |  | | |  |  | |
| **9HPT (s) - average** | **209** |  | **21** | | **19** | | **25** | | **26** | | **11** | **24** | | **21** | | **33** | | **29** | | **14** | | **21** | | **19** | | **24** | | | **22** | | **6** | | | <0.001 | <0.001 | |
| MIRS I | 21 | 33% | 21 | | 19 | | 23 | | 20 | | 2 | 22 | | 19 | | 23 | | 21 | | 2 | | 19 | | 17 | | 21 | | | 19 | | 2 | | | 0.03 |  | |
| MIRS II | 58 | 62% | 20 | | 19 | | 24 | | 22 | | 8 | 22 | | 19 | | 24 | | 23 | | 11 | | 20 | | 18 | | 22 | | | 21 | | 6 | | | *ns* |  | |
| MIRS III | 45 | 44% | 23 | | 21 | | 29 | | 28 | | 14 | 26 | | 22 | | 38 | | 32 | | 18 | | 23 | | 21 | | 24 | | | 23 | | 3 | | | 0.04 |  | |
| MIRS IV | 70 | 57% | 24 | | 20 | | 28 | | 26 | | 8 | 28 | | 25 | | 38 | | 31 | | 9 | | 21 | | 20 | | 25 | | | 22 | | 3 | | | <0.001 |  | |
| MIRS V | 15 | 40% | 29 | | 24 | | 53 | | 38 | | 19 | 40 | | 29 | | 53 | | 44 | | 19 | | 23 | | 22 | | 24 | | | 29 | | 17 | | | *ns* |  | |
| **adult onset phenotype** | **169** | 54% | **23** | | **20** | | **28** | | **26** | | **12** | **25** | | **20** | | **30** | | **27** | | **9** | | **21** | | **19** | | **23** | | | **22** | | **7** | | | <0.001 |  | |
| **late-onset phenotype** | **41** | 41% | **21** | | **19** | | **23** | | **22*** | | **8** | **22** | | **19** | | **24** | | **24***** | | **10** | | **20** | | **18** | | **23** | | | **21** | | **3** | | | *ns* |  | |
|  |  |  |  | |  | |  | |  | |  |  | |  | |  | |  | |  | |  | |  | |  | | |  | |  | | |  |  | |
| **9HPT (s) - best** | **209** |  | **21** | | **19** | | **25** | | **24** | | **10** | **23** | | **19** | | **31** | | **27** | | **12** | | **20** | | **18** | | **23** | | | **21** | | **6** | | | <0.001 | <0.001 | |
| MIRS I | 21 | 33% | 19 | | 18 | | 20 | | 19 | | 2 | 20 | | 18 | | 21 | | 20 | | 2 | | 19 | | 17 | | 20 | | | 18 | | 2 | | | *ns* |  | |
| MIRS II | 58 | 62% | 19 | | 18 | | 22 | | 20 | | 6 | 20 | | 18 | | 23 | | 21 | | 6 | | 19 | | 17 | | 20 | | | 20 | | 6 | | | *ns* |  | |
| MIRS III | 45 | 44% | 22 | | 19 | | 27 | | 26 | | 13 | 25 | | 19 | | 34 | | 29 | | 17 | | 21 | | 19 | | 24 | | | 22 | | 3 | | | *ns* |  | |
| MIRS IV | 70 | 57% | 23 | | 19 | | 26 | | 24 | | 7 | 27 | | 23 | | 34 | | 29 | | 8 | | 20 | | 19 | | 24 | | | 21 | | 3 | | | <0.001 |  | |
| MIRS V | 15 | 40% | 25 | | 22 | | 41 | | 34 | | 16 | 37 | | 25 | | 41 | | 38 | | 16 | | 22 | | 18 | | 23 | | | 27 | | 16 | | | *ns* |  | |
| **adult onset phenotype** | **169** | 54% | **21** | | **19** | | **26** | | **24** | | **11** | **24** | | **19** | | **28** | | **25** | | **8** | | **19** | | **18** | | **22** | | | **21** | | **6** | | | <0.001 |  | |
| **late-onset phenotype** | **41** | 41% | **20** | | **18** | | **23** | | **21***** | | **5** | **21** | | **19** | | **23** | | **22***** | | **6** | | **19** | | **17** | | **20** | | | **20** | | **3** | | | *ns* |  | |
|  |  |  |  | |  | |  | |  | |  |  | |  | |  | |  | |  | |  | |  | |  | | |  | |  | | |  |  | |
| **9HPT (s) non-dominant** | **208** | 50% | **24** | | **21** | | **29** | | **27** | | **10** | **26** | | **22** | | **36** | | **31** | | **13** | | **23** | | **20** | | **25** | | | **23** | | **5** | | | <0.001 | <0.001 | |
| **SARA (score)** | **202** |  | **4** | | **2** | | **8** | | **5** | | **5** | **5** | | **2** | | **9** | | **6** | | **5** | | **4** | | **2** | | **8** | | | **5** | | **4** | | | 0.4 | 0.3 | |
| MIRS I | 22 | 32% | 1 | | 0 | | 2 | | 1 | | 1 | 2 | | 0 | | 2 | | 1 | | 1 | | 1 | | 0 | | 3 | | | 1 | | 2 | | | *ns* |  | |
| MIRS II | 59 | 58% | 2 | | 1 | | 3 | | 3 | | 3 | 2 | | 1 | | 4 | | 3 | | 3 | | 2 | | 1 | | 3 | | | 3 | | 4 | | | *ns* |  | |
| MIRS III | 42 | 45% | 6 | | 3 | | 8 | | 6 | | 3 | 6 | | 3 | | 8 | | 5 | | 3 | | 6 | | 4 | | 9 | | | 6 | | 4 | | | *ns* |  | |
| MIRS IV | 64 | 61% | 7 | | 4 | | 10 | | 7 | | 4 | 8 | | 5 | | 11 | | 8 | | 4 | | 7 | | 4 | | 9 | | | 7 | | 4 | | | *ns* |  | |
| MIRS V | 15 | 40% | 14 | | 10 | | 16 | | 13 | | 5 | 15 | | 12 | | 15 | | 14 | | 5 | | 12 | | 10 | | 16 | | | 12 | | 5 | | | *ns* |  | |
| **adult onset phenotype** | **162** | 55% | **6** | | **2** | | **9** | | **6** | | **5** | **4** | | **2** | | **8** | | **5** | | **4** | | 4 | | 2 | | 8 | | | 5 | | 4 | | | *ns* |  | |
| **late-onset phenotype** | **40** | 40% | **2** | | **0** | | **3** | | **2***** | | **2** | **2** | | **0** | | **4** | | **2***** | | **3** | | **2** | | **0** | | **3** | | | **2***** | | **14** | | | *ns* |  | |
|  |  |  |  | |  | |  | |  | |  |  | |  | |  | |  | |  | |  | |  | |  | | |  | |  | | |  |  | |
| **Knee extensors QMT (lb)** | **212** |  | **44** | | **30** | | **62** | | **46** | | **20** | **55** | | **39** | | **68** | | **54** | | **20** | | **36** | | **26** | | **49** | | | **38** | | **17** | | | <0.001 | <0.001 | |
| MIRS I | 22 | 32% | 66 | | 48 | | 74 | | 61 | | 19 | 70 | | 63 | | 78 | | 67 | | 17 | | 41 | | 35 | | 64 | | | 48 | | 15 | | | 0.02 |  | |
| MIRS II | 59 | 61% | 54 | | 40 | | 65 | | 54 | | 17 | 64 | | 55 | | 72 | | 65 | | 16 | | 49 | | 36 | | 56 | | | 47 | | 15 | | | <0.001 |  | |
| MIRS III | 46 | 43% | 45 | | 34 | | 62 | | 47 | | 17 | 55 | | 36 | | 63 | | 52 | | 15 | | 38 | | 31 | | 47 | | | 41 | | 18 | | | 0.04 |  | |
| MIRS IV | 70 | 57% | 31 | | 25 | | 45 | | 37 | | 19 | 43 | | 31 | | 62 | | 47 | | 20 | | 28 | | 19 | | 36 | | | 29 | | 15 | | | <0.001 |  | |
| MIRS V | 15 | 40% | 27 | | 14 | | 44 | | 30 | | 16 | 35 | | 14 | | 50 | | 35 | | 18 | | 21 | | 16 | | 27 | | | 22 | | 8 | | | *ns* |  | |
| **adult onset phenotype** | **171** | 52% | **40** | | **29** | | **60** | | **44** | | **20** | **56** | | **37** | | **68** | | **55** | | **20** | | **35** | | **25** | | **48** | | | **37** | | **17** | | | <0.001 |  | |
| **late-onset phenotype** | **41** | 41% | **56** | | **40** | | **69** | | **56***** | | **17** | **50** | | **49** | | **71** | | **47*** | | **15** | | **50** | | **35** | | **49** | | | **47**** | | **15** | | | 0.007 |  | |
|  |  |  |  | |  | |  | |  | |  |  | |  | |  | |  | |  | |  | |  | |  | | |  | |  | | |  |  | |
| **Hip flexors QMT (lb)** | **201** |  | **33** | | **24** | | **42** | | **33** | | **13** | **39** | | **28** | | **48** | | **39** | | **13** | | **27** | | **20** | | **34** | | | **28** | | **10** | | | <0.001 | <0.001 | |
| MIRS I | 19 | 37% | 42 | | 37 | | 53 | | 44 | | 11 | 70 | | 63 | | 78 | | 48 | | 10 | | 30 | | 27 | | 37 | | | 32 | | 7 | | | 0.005 |  | |
| MIRS II | 59 | 61% | 38 | | 28 | | 46 | | 38 | | 13 | 64 | | 55 | | 72 | | 45 | | 12 | | 33 | | 25 | | 43 | | | 33 | | 12 | | | 0.001 |  | |
| MIRS III | 43 | 44% | 35 | | 26 | | 42 | | 35 | | 11 | 55 | | 36 | | 63 | | 38 | | 11 | | 33 | | 23 | | 36 | | | 30 | | 9 | | | 0.02 |  | |
| MIRS IV | 68 | 59% | 25 | | 20 | | 33 | | 27 | | 11 | 43 | | 31 | | 62 | | 33 | | 13 | | 22 | | 19 | | 28 | | | 23 | | 7 | | | <0.001 |  | |
| MIRS V | 12 | 42% | 20 | | 16 | | 31 | | 25 | | 12 | 35 | | 14 | | 50 | | 29 | | 13 | | 19 | | 17 | | 20 | | | 18 | | 3 | | | *ns* |  | |
| **adult onset phenotype** | **161** | 56% | **30** | | **23** | | **40** | | **32** | | **13** | **38** | | **27** | | **47** | | **39** | | **15** | | **28** | | **20** | | **34** | | | **28** | | **10** | | | <0.001 |  | |
| **late-onset phenotype** | **40** | 43% | **40** | | **34** | | **76** | | **39**** | | **13** | **41** | | **35** | | **49** | | **42** | | **12** | | **35** | | **25** | | **44** | | | **34**** | | **114** | | | 0.05 |  | |
|  |  |  |  | |  | |  | |  | |  |  | |  | |  | |  | |  | |  | |  | |  | | |  | |  | | |  |  | |
| **Ankle dorsi-flexors QMT (lb)** | **188** |  | **23** | | **15** | | **35** | | **26** | | **13** | **27** | | **17** | | **40** | | **29** | | **15** | | **21** | | **14** | | **33** | | | **23** | | **11** | | | 0.003 | 0.05 | |
| MIRS I | 22 | 32% | 43 | | 30 | | 54 | | 42 | | 14 | 54 | | 37 | | 56 | | 47 | | 12 | | 29 | | 20 | | 39 | | | 30 | | 10 | | | 0.004 |  | |
| MIRS II | 59 | 61% | 31 | | 22 | | 39 | | 31 | | 12 | 36 | | 27 | | 45 | | 35 | | 12 | | 30 | | 19 | | 34 | | | 28 | | 11 | | | 0.021 |  | |
| MIRS III | 41 | 44% | 19 | | 15 | | 27 | | 21 | | 11 | 19 | | 12 | | 26 | | 20 | | 11 | | 20 | | 16 | | 28 | | | 23 | | 11 | | | *ns* |  | |
| MIRS IV | 58 | 62% | 18 | | 13 | | 24 | | 19 | | 9 | 20 | | 12 | | 28 | | 21 | | 9 | | 17 | | 13 | | 22 | | | 19 | | 9 | | | *ns* |  | |
| MIRS V | 8 | 63% | 13 | | 6 | | 17 | | 14 | | 10 | 13 | | 5 | | 36 | | 18 | | 16 | | 13 | | 6 | | 16 | | | 12 | | 6 | | | *ns* |  | |
| **adult onset phenotype** | **147** | 58% | **21** | | **14** | | **30** | | **23** | | **14** | **21** | | **15** | | **32** | | **25** | | **13** | | **20** | | **15** | | **32** | | | **22** | | **11** | | | *ns* |  | |
| **late-onset phenotype** | **41** | 41% | **37** | | **27** | | **48** | | **36***** | | **15** | **41** | | **28** | | **55** | | **40***** | | **16** | | **32** | | **18** | | **42** | | | **30**** | | **13** | | | 0.01 |  | |
| QMT: quantitative muscle testing (best score of three) | | | | | | | | | | | | | | | | | |  | |  | |  | |  | |  | | |  | |  | | |  |  | |
| *average scores between phenotype subgroups differ with a significance <0.05 | | | | | | | | | | | | | | |  | |  | |  |  |  | |  | |  | |  | | | |  |  | | | |  |
| **average scores between phenotype subgroups differ with a significance <0.01 | | | | | | | | | | | | | | |  | |  | |  |  |  | |  | |  | |  | | | |  |  | | | |  |
| *** average scores between phenotype subgroups differ with a significance <0.001 | | | | | | | | | | | | | | |  | |  | |  |  |  | |  | |  | |  | | | |  |  | | | |  |
| *ns* not significant (i.e. >0.05) | | | |  | |  | |  | |  |  | |  |  | |  | |  | |  | |  | |  | |  | |  | |  | | |  | |  | |
